# One-year update on physical activity and smartphone addiction in university students: A systematic review of novel research

**DOI:** 10.1016/j.pmedr.2025.103178

**Published:** 2025-07-20

**Authors:** Neha Pirwani, Attila Szabo

**Affiliations:** a Institute of Health Promotion and Sport Sciences, Faculty of Psychology and Education, ELTE Eötvös Loránd University, Budapest, Hungary; b Doctoral School of Education, Faculty of Psychology and Education, ELTE Eötvös Loránd University, Budapest, Hungary; c Department of Psychology and Health Management, Faculty of Health and Sport Sciences, Széchenyi István University Győr, Hungary

**Keywords:** Behavioral addiction, Dependence, Physical activity, Internet, Mobile phone

## Abstract

**Objectives:**

Smartphone addiction is a growing health concern, especially among university students. This updated review expands our 2024 review by synthesizing recent empirical findings on the relationship between physical activity and smartphone addiction among university students.

**Methods:**

Following PRISMA guidelines, a thorough search was conducted in PubMed, SSRN, Oxford Research Archive, JSTOR, and Google Scholar. The quality of studies was evaluated with the Mixed Methods Appraisal Tool.

**Results:**

Sixteen studies published between January 1, 2024, and March 31, 2025, met the inclusion criteria: 14 cross-sectional, one randomized controlled trial, and one longitudinal. All emerged from Asia. Most found an inverse relationship between physical activity and smartphone addiction. Experimental and longitudinal studies indicated that structured physical activity could reduce smartphone addiction symptoms over time. However, the predominantly correlational designs warrant cautious interpretation. Still, the directionally consistent average effect size was moderate to large (Cohen's *d =* ≈ −0.62), highlighting physical activity's protective role.

**Conclusions:**

Regular physical activity may prevent or reduce smartphone addiction in university students. However, future studies should employ longitudinal designs, use objective measures, and incorporate qualitative validation. This review reinforces our earlier findings and supports the considerable inverse relationship between physical activity and smartphone addiction.

## Highlights

• Sixteen new studies examined the link between smartphone use and physical activity
• A moderate to large average effect size was found (Cohen's *d* = −0.62)
• Self-control and self-esteem mediate the smartphone–exercise relationship
• Hedonic versus utilitarian smartphone use may influence exercise behavior
• Gut microbiota may also play a role in smartphone and exercise associations

## 1 Introduction

Since the release of the first iPhone in 2007, smartphones have transitioned from luxury items to indispensable tools of modern life. Today, mobile phone subscriptions outnumber the global population (World Economic Forum, 2023). As of 2025, global smartphone usage is projected to reach 7.49 billion users, while the world population is approximately 8.09 billion (Statista., 2023; United States Census Bureau, 2025). This suggests that nearly everyone owns a smartphone, except for the very young. The COVID-19 pandemic significantly deepened this dependence, as individuals relied on smartphone applications such as WhatsApp, Zoom, and online marketplaces to maintain daily routines and social connections while confined to their homes (Ausman, 2021). Although smartphones provide extensive utility and convenience across various aspects of life, their use can become excessive and potentially harmful.

Despite contributing positively to global health through innovation (Ye, 2020), the overuse of smartphones raises concerns, particularly regarding problematic use or addiction. One emerging issue is smartphone addiction, which has drawn attention for its links to decreased physical activity and its resemblance to other behavioral addictions, including internet gaming disorder and gambling (Lin et al., 2017). Core symptoms of smartphone addiction include compulsive overuse, loss of control, diminished productivity, functional impairment, and withdrawal symptoms during non-use (Duke and Montag, 2017; Lin et al., 2014; Rozgonjuk et al., 2020).

A growing body of research suggests that individuals with lower self-control are particularly vulnerable to problematic smartphone use. According to the Uses and Gratifications Theory (Ruggiero, 2000), this dependency may arise from attempts to fulfill internal psychological needs, especially among university students, who frequently use their phones to manage emotional or cognitive states. While these psychological needs vary individually, they often result in difficulty disengaging from smartphone use (Liu et al., 2022). Common traits observed among students with high smartphone addiction include frequent boredom, diminished impulse control, poor planning ability, and discomfort or disorientation when separated from their phones (Ding et al., 2021; Panza et al., 2017; Pengpid and Peltzer, 2019). In addition, smartphone addiction has been associated with adverse outcomes such as poor emotional regulation, increased anxiety, sleep disturbances, reduced well-being (Cho and Lee, 2017; Gökçearslan et al., 2016), social withdrawal, loneliness, academic decline, and even suicidal thoughts (Bian and Leung, 2014; Kuss and Griffiths, 2011).

Furthermore, individuals addicted to the internet on smartphones often engage in minimal physical activity and tend to neglect their overall health (Kim, 2013). This behavior can lead to physical complications such as carpal tunnel syndrome, poor posture, migraines, back pain, eye strain, disrupted eating habits, sleep deprivation, dry eyes, and even hygiene neglect (Wieland, 2005). Over time, these problems may disrupt hormone regulation, weaken the immune system, and affect cardiovascular and digestive health (Wieland, 2005). Physically, smartphone overuse may also contribute to issues such as headaches, reduced attention span, and impaired memory (Khan, 2008). One critical health implication is its association with sedentary behavior. For instance, university students with lower physical activity levels are significantly more likely to engage in prolonged smartphone use (Grimaldi-Puyana et al., 2020). Inadequate physical activity itself poses numerous risks to both physical and mental health (Carson et al., 2016). Globally, inactivity is alarmingly prevalent: over 80 % of Southeast Asian youth are physically inactive (Peltzer and Pengpid, 2016), and the World Health Organization (2018) estimates that 81 % of adolescents, 23 % of young adults, and 55 % of older adults do not meet recommended physical activity levels.

Several studies have reported a notably high prevalence of smartphone addiction among university students. For instance, Buctot et al. (2020) observed that 62.6 % of their participants met the criteria for smartphone addiction, highlighting the widespread nature of this issue in student populations (Al-Barashdi, 2015; Chen et al., 2017). Given the intersecting concerns of rising smartphone dependence and declining physical activity, researchers have increasingly explored the relationship between the two. Physical activity is crucial in offsetting the sedentary lifestyle often associated with heavy digital use among younger populations (Park et al., 2020). It supports emotional resilience and serves as a constructive way to manage stress, counteracting the negative impacts of extended screen exposure and promoting mental well-being (Lai et al., 2025). Structured physical activity, such as exercise or sports, has been related to psychological benefits that could potentially mitigate addiction-related behaviors (Surawska, 2017). Since addictions are primarily psychological (Dodes, 2009), promoting mental well-being through physical activity may offer a preventive strategy. For example, a 12-week intervention led to sustained improvements in psychological functioning (DiLorenzo et al., 1999), findings echoed in later work (Stathopoulou et al., 2006). Such evidence suggests that physically active individuals may be less susceptible to smartphone addiction and enjoy better psychological resilience (Glenister, 1996; Liu et al., 2022).

The current review focuses specifically on university students, a population identified as especially vulnerable to smartphone addiction (Csibi et al., 2019). *Physical activity* is defined here as *planned* sports or exercises practiced for health, leisure, mastery, or competition (Caspersen et al., 1985), consistent with the definition used in our previous review. This definition enables a wide-ranging review of both competitive and leisure activities. Meanwhile, smartphone addiction is again broadly conceptualized as the risk or predisposition to ill-patterned smartphone-based application (s) use, consistent with the addiction trajectory outlined in the pathway model (Billieux et al., 2015). This definition excludes studies concerning the model's risky or antisocial pathways. Additionally, studies that use questionnaires assess risk and/or predisposition without providing clinical diagnoses (Szabo et al., 2015). Therefore, we consider problematic smartphone use and dependence as warning signs of potentially addictive smartphone use, while recognizing that current questionnaire-based studies only measure "risk" levels that have no diagnostic value.

This updated systematic review builds on our previous review (Pirwani and Szabo, 2024), which synthesized the literature on the association between smartphone addiction and physical activity among university students. The original review provided foundational insights by mapping the emerging evidence and conceptual frameworks linking these variables. The widespread interest in the intersection of smartphone addiction and physical activity continues to grow, underscoring the timeliness of this update. Within a year, our original review garnered over 140 reads and 22 academic citations, reflecting its relevance in academic and applied contexts. Despite this increasing attention, no recent review has consolidated studies published after 2023, creating a gap in synthesized knowledge. This updated review aims to address this void by analyzing newly published empirical research, thereby providing an up-to-date synthesis of the evolving evidence base and offering refined insights into the dynamics of smartphone addiction and physical activity among university students.

## 2 Methods

### 2.1 Inclusion and exclusion criteria

This literature review update considers studies published in peer-reviewed journals and written in the English language. In this paper, we refrain from reanalyzing previously reviewed studies and instead focus exclusively on new peer-reviewed studies published from January 1, 2024, to March 31, 2025. This selective inclusion enables a clearer emphasis on recent findings, positioning the current review as a complement to the original and providing a more comprehensive and up-to-date understanding of the topic. One study by Kumar et al. (2024) was excluded because it had already been reviewed in our original paper (Pirwani and Szabo, 2024).

Research designs include cross-sectional studies, longitudinal analyses, intervention-based research, and randomized controlled trials. As previously indicated, the population of interest consists of university students, typically young adults, who are among the age group most susceptible to smartphone addiction (Csibi et al., 2019). This review focuses on studies that explicitly investigate the relationship between physical activity and smartphone addiction.

Studies were excluded if they were not published in English or if they appeared in non-academic sources such as newspapers or magazines. Additional exclusions encompass commentaries, dissertations, conference abstracts or proceedings, editorials, opinion or methodological papers, pilot studies, books, book chapters, articles lacking a clear methodological framework, and studies available only in abstract form (see Table 1).

**Table 1:** Eligibility Criteria for Including Studies in the Systematic Review of Research on Smartphone Addiction and Physical Activity Among University Students (January 01, 2024–March 31, 2025, Various Countries).

Inclusion Criteria	Exclusion Criteria
Articles published in English	Abstracts
Papers in peer-reviewed journals	Books (or chapters)
Measures smartphone addiction	Conference Proceedings
Assesses any form of physical activity	Dissertations
Examines university/college students	Editorials, commentaries
Articles published from January 01, 2024	Literature reviews
	Methodological papers
	Short reports

### 2.2 Search strategy

A serial search was conducted across five electronic databases: PubMed, Social Sciences Research Network (SSRN), Oxford Research Archive (ORA), JSTOR, and Google Scholar. Following screening the first four databases, Google Scholar was searched last to reduce redundancy and limit duplicate records. This order of operations enhanced the efficiency of the review process. Gehanno et al. (2013) argue that Google Scholar provides access to a broader range of academic materials. Its demonstrated ability to retrieve 100 % of the studies in certain systematic reviews highlights its value, particularly in capturing research that may be overlooked by traditional databases (Gehanno et al., 2013).

To ensure thoroughness, the reference lists of relevant studies were manually reviewed for additional sources that fit the eligibility criteria. The search strategy was organized around three thematic keyword clusters related to smartphones, physical activity, and university students. Table 2 provides an overview of the search terms employed. Boolean operators were used where appropriate to refine the results and enhance the precision of the search process.

**Table 2:** Search Terms and Boolean Operators Used in Literature Search for Studies Examining the Relationship Between Smartphone Addiction and Physical Activity in University Students (January 01, 2024–march 31, 2025).

	AND		AND	
Smartphone addict*, OR Smartphone depend*, OR Smartphone overuse, OR Compulsive smartphone* use OR Excessive smartphone* use OR Problematic smartphone use, OR Exaggerated smartphone* use OR Mobile phone addict*, OR Mobile phone* depend*, OR Mobile phone overuse, OR Compulsive mobile phone use OR Excessive mobile phone use OR Problematic mobile phone use OR Exaggerated mobile phone use		Physical Activity, OR Exercise*, OR Sport*, OR Training		University student*, OR Freshmen, OR Undergraduate* OR Graduate*, OR Major*

*Note:* The wild card (*) extends the search to any ending of the word trunk.

### 2.3 Search outcome

This review was conducted in accordance with the Preferred Reporting Items for Systematic Reviews and Meta-Analyses (PRISMA) framework (Liberati et al., 2009), which provides a standardized structure for transparent and comprehensive reporting of systematic reviews. A total of 193 records were retrieved from the selected databases, with the following distribution: PubMed (60), Social Science Research Network (4), Oxford Research Archive (7), JSTOR (15), and Google Scholar (107). A manual search identified one additional record, including references cited in the initially retrieved articles.

**Fig. 1:**
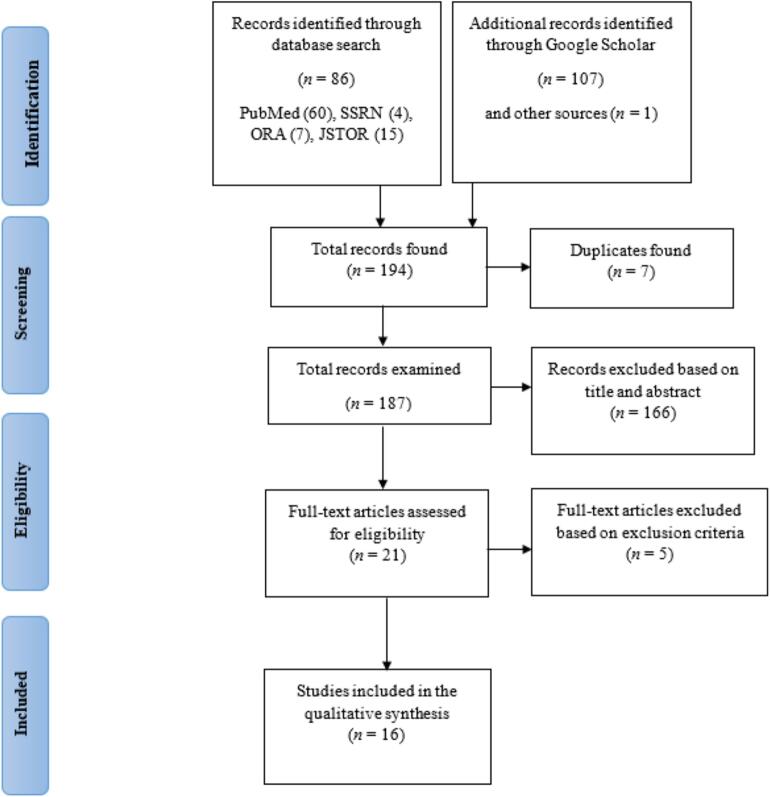
PRISMA (Preferred Reporting Items for Systematic Reviews and Meta-Analyses) Flow Diagram Illustrating the Selection Protocol of Studies Included in the Systematic Review on Smartphone Addiction and Physical Activity in University Students (January 01, 2024–March 31, 2025).

After eliminating seven duplicates, a pool of 187 unique articles was subjected to title and abstract screening. This preliminary review led to 21 full-text articles deemed potentially relevant. Further eligibility checks excluded five articles: one focused on smartphone applications promoting physical activity rather than examining the relationship between physical activity and smartphone addiction, and four investigated age groups that did not align with the defined population of interest. Ultimately, 16 full-text studies met all inclusion criteria and were incorporated into this review (see Fig. 1).

### 2.4 Quality assessment

As in the original review, the Mixed Methods Appraisal Tool (MMAT) was used to evaluate the methodological quality and potential bias of the studies selected for inclusion. This tool has been recognized as efficient and robust for systematic review contexts (Pace et al., 2012; Hong et al., 2018). The assessment begins with two initial screening questions that determine whether the research questions are clearly stated and whether the data collected adequately address those objectives.

Following the screening phase, each study is evaluated using five questions corresponding to its research design. These questions assess aspects such as the appropriateness of the sampling strategy, the sample's representativeness, the appropriateness of measurement tools, the potential for nonresponse bias, and the adequacy of the statistical analysis. Responses are recorded as ‘Yes,’ ‘No,’ or ‘Uncertain’ depending on the clarity and rigor demonstrated in each study (Hong et al., 2018).

In this updated review, two independent reviewers conducted the quality appraisal process. They agreed on the preliminary screening questions and all five subsequent research design-specific items. The evaluation determined whether the studies' focus was on the link between smartphone addiction and physical activity, considering the study sample, methods, measures, and analytical techniques. Based on this appraisal, all 16 studies were deemed methodologically sound and were included in the review without any concerns regarding quality or bias.

### 2.5 Data extraction

After completing the MMAT quality appraisal and confirmation of the validity of the data, relevant information from each study was systematically extracted and compiled into Table 3. This structured extraction process captured essential study characteristics, including author names, year of publication, research geographic location, and participants' demographic details. In addition, data related to smartphone addiction and physical activity measures were documented, along with the statistical analyses performed and the principal findings reported in each study.

**Table 3:** Summary of Study Characteristics and Main Findings on the Association Between Physical Activity and Smartphone Addiction in University Students (Published January 01, 2024–March 30, 2025, Various Countries).

**Author & year**	**Country**	**Study Type**	**Sample**	**Measurement**	**Tests and effects**	**Estimated effect size in Cohen's *d* ≈**	**Main findings**
1. Aligul & Tolukan (2024)	Turkey	CS	461 University students (248 M, 213 F) Age range = 18 to 25 years	MSPPA, SAS (Turkish version)	Independent samples *t*-test, One-way ANOVA, Pearson correlation	−0.27 (small effect)	Smartphone addiction and physical activity motivation were negatively correlated. Smartphone addiction was higher in the 18–21 vs. the 22–25 age group; PA motivation was higher in females.
2. Anwar et al. (2024)	Pakistan	CS	330 University students (165 university-level athletes; 165 non-athletes M/F not specified) Age range = 19 to 25 years	IPAQ-SV, SAS	Independent-samples *t*-tests with preferred	−1.22 (large effect)	Non-athletes exhibited significantly higher smartphone addiction scores than athletes, suggesting that physical activity may serve a protective role against smartphone addiction.
3. Ke et al. (2024)	China	CS	608 College students (288 M; 320 F) Mean age = 20.27 ± 1.69 years	PARS-3, MPATS, SES	Correlation analysis, Hierarchical regression analysis, Mediating effect analysis	−0.25 (small effect)	Physical activity reduced smartphone addiction directly and indirectly by enhancing self-esteem.
4. Kumban et al. (2025)	Thailand	CS	120 University students (43 M; 77 F) Mean age = 20.44 ± 1.31 years	GPAQ, SAS-SV (Thai version)	Mann-Whitney *U* test, Kruskal-Wallis H test, Chi-square tests, Spearman's rank correlation	No effect size reported	No significant correlation was found between physical activity and smartphone addiction. Both smartphone addicted and non-addicted groups had >8 h daily screen time
5. Lai et al. (2025)	China	CS	3506 College students (1743 M; 1763 F) Mean age = 19 years	PARS-3, SAS-SV (Chinese version)	Multivariate logistic regression, Stratified and interaction analyses	−0.20 (small effect)	Physical activity and higher exercise intensity were negatively associated with smartphone addiction. Smartphone addiction prevalence was lower in physically active students.
6. Liang and Tang (2025)	China	CS	900 college students (M/F not specified) Age range not specified	PARS-3, MPAI (Chinese version), SCSS, Simplified SCS	Pearson correlation, SEM, Mediation analysis	−0.34 (small effect)	Physical activity was negatively associated with SA; this link was partially mediated by positive coping and moderated by self-control.
7. Liu et al. (2024)	China	CS	697 College students (179 M; 518 F) Mean age = 19.22 ± 1.00 years	PARS-3, SCS, Mobile Phone Dependence Questionnaire	Pearson correlation, Regression analysis, Mediation and moderation analyses	−0.31 (small effect)	Physical activity was positively linked to self-control, which in turn was negatively associated with mean physical activity, suggesting that physical activity may indirectly reduce sedentary behavior in students.
8. Meng et al. (2025)	China	CS	4562 College students (3570 M; 992 F) Mean age = 19.59 ± 1.21 years	MPATS, PARS-3	SEM	−2.16 (very large effect)	Physical activity was negatively associated with smartphone addiction and may help alleviate it.
9. Su et al. (2024)	China	CS	1315 College students (623 M; 692 F) Age range = 16 to 33 years	SABAS, Self-administered questionnaire for PA	Generalized Linear Models, Various correlation analyses	Frequency = −0.33 Duration = −0.32 (small effects)	Higher frequency and duration of nighttime physical activity were linked to lower smartphone addiction levels and reduced smartphone use before sleep
10. Wang (2025)	China	CS	413 University students (208 M; 205 F) Mean age= 20.59 ± 1.17 years	PARS-3, MPAI, SCS, CD-RISC	Pearson's correlation, Regression analysis using mediation models	−0.99 (large effect)	Physical activity was negatively associated with smartphone addiction and also reduced it through the chain-mediated effects of self-control and resilience
11. Wang et al. (2024a)	China	CS	274 College students (146 M; 128 F) Mean age = 20.31 ± 1.29 years	IPAQ-SV, SAS	Pearson correlation, Chained mediation effect tests	−0.46 (medium effect)	Physical activity was negatively associated with smartphone addiction, suggesting that students who engaged in more physical activity tended to have lower smartphone addiction.
12. Wang et al. (2024b)	China	Longitudinal	414 College students (197 M; 217 F) Mean age = 20.60 ± 0.83 years	PARS-3, MPATS, SCS	Mann-Whitney U test, Correlation analysis, Cross-lagged relationship model	−0.48 (medium effect)	Physical activity negatively influenced smartphone addiction both directly and indirectly through improved self-control.
13. Yin et al. (2024)	China	CS	2274 College students (743 M; 1531 F) Mean age = 19.18 ± 1.02	PARS-3, SAS-SV, SCS (Chinese version),	Standard method bias tests, Correlation analysis, Regression analysis, Chi-square tests	−0.77 (medium-large effect)	Physical activity was negatively associated with smartphone addiction and positively linked to self-control, indicating physical activity may reduce smartphone addiction directly and indirectly.
14. Zeren et al. (2024)	Turkey	CS	46 University students (29 F; 17 M) Mean age = 21.02 ± 1.47	SAS-SF, IPAQ-SF, Exercise Benefits/Barriers Scale	Pearson/Spearman correlation, Regression analysis, Independent samples t-test/Mann-Whitney *U* test	−0.13 to −0.17 (negligible effects)	No significant association was found between smartphone addiction and physical activity participation or maximal exercise capacity metrics
15. Zhang et al. (2024)	China	EXP (RCT)	90 College students (36 M; 54 F) Mean age = 20.11 ± 0.644	SAS-SV, Aerobic exercise (AE) group, Tai Chi Chuan (TCC) group, Wait-list control (WLC), GSES, Fatigue Scale-14	GEE analysis, Spearman correlation	AE = −1.69 (large effect) TCC = −1.16 (large effect)	Aerobic exercise and Tai Chi reduced problematic mobile phone use, showing that physical activity can be an effective intervention for smartphone addiction.
16. Zhu et al. (2024)	China	CS	4670 College students (1714 M; 2956 F) The age range was not specified	MPATS, PARS-3, PSQI	Chi-square test, One-way ANOVA, Correlation analysis, Linear regression analysis	−0.20 (small effect)	Smartphone addiction was negatively associated with physical activity. Physical activity was a moderator in the relationship between smartphone addiction and sleep quality.

Note: Table acronyms in alphabetical order: CD-RISC = Connor-Davidson Resilience Inventory Scale Chinese; GEE = Generalized Equation Analysis; GPAQ = Global Physical Activity Questionnaire; GSES = General Self-Efficacy Scale; IPAQ-SV = International Physical Activity Questionnaire-Short Version; MPAI = Mobile Phone Addiction Index; MPATS = Mobile Phone Addiction Tendency Scale; MSPPA = Motivation Scale for Participation in Physical Activity; PARS-3 = Physical Activity Rating Scale-3; PSQI = Pittsburgh Sleep Quality Index; SAS = Smartphone Addiction Scale; SCSS = Simple Coping Style Scale; SCS = Self-Control Scale; SEM = Structural Equation Modeling; SES = Self Esteem Scale. The references for the studies in this Table are listed in Supplementary Table. 4.

## 3 Results

Based on the converted effect sizes presented in Table 3 (and supplementary Table 4), the average effect size across all included studies was approximately Cohen's *d* ≈ −0.617, indicating a medium-to-large inverse association between smartphone addiction and physical activity in university students. Furthermore, the RCT intervention study yielded a large effect size. Table 3 summarizes the results. The majority, 14 of the 16 included studies, employed cross-sectional designs. At the same time, one utilized a three-wave longitudinal cross-lagged panel model, while the other adopted an experimental, randomized controlled trial (RCT) approach. Except for two studies conducted in Turkey, one in Pakistan, and one in Thailand, the remaining studies were all carried out in China, including both the intervention-based and longitudinal investigations.

Nine of the studies were hypothesis-driven, whereas the remaining seven were exploratory. Five validated instruments were used across studies to measure smartphone addiction. Physical activity was assessed through four different scales in the cross-sectional studies, with one study using a self-developed questionnaire that demonstrated acceptable internal consistency (Cronbach's alpha >0.70).

Sample sizes varied considerably. Cross-sectional studies ranged from a minimum of 46 participants (Zeren et al., 2024) to a maximum of 4670 participants (Zhu et al., 2024). The RCT involved 90 participants (Zhang et al., 2024), while the longitudinal study was based on a sample of 414 individuals (Wang et al., 2024b).

To enhance comparability across studies, all available reported effect sizes were converted into a standard unit (Cohen's *d*), where sufficient statistical information was provided. These values are included in a dedicated column in Table 3, alongside the original reported effects. While this conversion provides a more straightforward overview of the strength and direction of associations, the diversity in research design, sample characteristics, and measurement tools across studies should be considered when interpreting the results.

### 3.1 Smartphone addiction and physical activity: Summary of cross-sectional studies

Most recent cross-sectional studies confirmed a negative relationship between smartphone addiction and physical activity, in line with earlier findings. Aligül and Tolukan (2024) found that smartphone addiction was inversely related to physical activity motivation, especially among younger students (18–21). Anwar et al. (2024) reported lower smartphone addiction scores in university athletes compared to non-athletes, suggesting a protective effect of physical activity. Lai et al. (2025) observed an 11.3 % lower prevalence of smartphone addiction among active students, with a higher intensity associated with a lower risk. Similar negative associations were identified by Meng et al. (2025), Wang et al. (2024a), and Yin et al. (2024). However, Kumban et al. (2025) and Zeren et al. (2024) found no significant relationship, emphasizing the need to explore potential moderators. Overall, 14 of 16 studies supported an inverse relationship between physical activity and smartphone addiction.

### 3.2 Smartphone addiction and physical activity: Summary of intervention & longitudinal studies

Intervention and longitudinal studies provide strong evidence that structured physical activity helps reduce problem behaviors like SA. Zhang et al. (2024) demonstrated that aerobic exercise and Tai Chi Chuan lowered problematic mobile phone use and improved factors such as fatigue and gut microbiota. Similarly, Wang et al. (2024b) found that consistent physical activity over a year increased self-control, which then decreased future smartphone addiction. These results support the notion that structured exercise can alleviate symptoms of smartphone addiction and enhance overall well-being in university students.

### 3.3 Smartphone addiction, physical activity, and self-control

Several studies explicitly explored self-control's role as a mediator or moderator in the relationship between smartphone addiction and physical activity. Liang and Tang (2025) revealed that positive coping strategies partially mediated this relationship, with students who had greater self-control benefiting more significantly from physical activity. Liu et al. (2024), Wang (2025), and Wang et al. (2024b) confirmed that higher self-control, fostered by regular physical activity, led to a subsequent reduction in smartphone addiction. These studies highlight that physical activity can indirectly reduce smartphone addiction by strengthening students' self-control. Hence, providing further evidence that enhancing self-regulatory abilities can buffer against smartphone overuse.

### 3.4 Smartphone addiction, physical activity, and psychological well-being

Beyond self-control, studies show that physical activity has broader psychological benefits on smartphone addiction. Ke et al. (2024) found that physical activity reduced smartphone addiction both directly and indirectly by boosting self-esteem, while Su et al. (2024) demonstrated that evening physical activity decreased nighttime smartphone use and anxiety. Zhu et al. (2024) reported that regular physical activity moderated the negative impact of smartphone addiction on sleep quality. Wang (2025) identified resilience as a mediator in the relationship between physical activity and smartphone addiction, thereby reinforcing the mental health benefits of regular exercise. Together, these empirical findings point towards physical activity's potential to enhance psychological well-being and reduce both the risk and severity of smartphone addiction.

## 4 Discussion

This updated systematic review revisits the relationship between smartphone addiction and physical activity among university students by analyzing 16 additional studies published in 2024 and 2025, following the publication of our original paper (Pirwani and Szabo, 2024). Like the original review, the current update primarily includes cross-sectional studies, one intervention study, and one longitudinal study. Overall, 14 out of 16 studies confirm an inverse relationship between physical activity and smartphone addiction, indicating that higher physical activity levels are generally associated with fewer smartphone addiction symptoms.

The average effect size calculated across studies in this update (Cohen's d ≈ −0.62) is slightly weaker than in our original review (Pirwani and Szabo, 2024), which, although not explicitly reported at the time, yielded a slightly stronger average effect size (*d* ≈ −0.69). Both reviews confirm a consistent inverse relationship between physical activity and smartphone addiction. However, the earlier review included several intervention studies with considerable effect sizes, elevating the overall average. In contrast, based exclusively on newer studies published in 2024–2025, the present update still reveals a robust negative association, further supporting physical activity as a meaningful protective factor.

Building on previous evidence, recent studies emphasize important moderating and mediating factors. Liang and Tang (2025) and Wang (2025) expanded on mechanisms such as self-control, resilience, and psychological well-being (Guo et al., 2022; Xie et al., 2019; Zhao et al., 2022) in the relationship between physical activity and smartphone addiction. Exercise intensity also proved to be crucial: Lai et al. (2025) found that higher-intensity exercise was associated with lower smartphone addiction risk, echoing Fan et al. (2021), who demonstrated that 30 min of moderate aerobic activity improved NoGo accuracy and reaction time in students with SA. These findings suggest that physical activity may boost self-regulation, providing a pathway to reduce addictive behaviors. Supporting this, Lai et al. cited Cai et al. (2023), who reported that higher physical activity and intensity levels correlated with lower internet addiction, indicating similar benefits for smartphone use.

Intervention and longitudinal studies in this review strengthen the evidence base. Zhang et al. (2024) found that aerobic exercise and Tai Chi Chuan reduced problematic mobile phone use and improved gut microbiota. This supports earlier RCTs where 12 weeks of basketball and Baduanjin yielded similar reductions (Xiao et al., 2021). A year-long study by Wang et al. (2024b) confirmed the lasting effect of physical activity on self-control and reduced smartphone addiction, a pattern also observed by Zhao et al. (2024). These findings underscore the importance of structured exercise interventions and the use of both subjective and objective measures in future research.

Despite the overall robust findings, some studies reported null results. Kumban et al. (2025) and Zeren et al. (2024) found no significant link between physical activity and smartphone addiction, echoing earlier findings (Demirbilek and Minaz, 2020; Zhao et al., 2022). However, small samples (e.g., Zeren et al., *n* = 46) may limit the generalizability of the findings. Kumban et al. noted that their physically active sample also had high screen time (8 h or more per day). Both smartphone addiction and non-smartphone addiction groups met the World Health Organization's physical activity guidelines (≥150 min/week), suggesting physical activity and screen use may coexist. This paradox underscores the importance of considering the quantity, context, and timing of screen use in conjunction with the type of physical activity.

This updated review introduces new complexities that the original publication did not cover. Aligul and Tolukan (2024) reported that licensed student athletes scored higher on smartphone addiction despite high physical activity engagement. Findings by Drenowatz et al. (2019) and Schroeder et al. (2017) suggest that participation in club sports and membership in health clubs are associated with greater fitness and higher training levels, respectively. This suggests that high smartphone addiction scores in active individuals may not always indicate problematic use. Instead, their smartphone engagement might be purpose-driven, such as for performance tracking, training guidance, or team communication, rather than out of compulsive behavior. This distinction between hedonic and utilitarian smartphone use is essential for populations heavily involved in sports and fitness. Without this contextual detail, interpretations of smartphone addiction risk in active populations could be misleading or too broad. Future research should focus on differentiating between the types and motivations of smartphone use to more accurately assess their potential for problematic use.

Another preliminary and exploratory contribution comes from Zhang et al. (2024), who reported a significant negative association between specific gut microbiota (Bacteroidaceae, Bacteroides, Alistipes) and smartphone addiction scores. As this finding is based on a single RCT with a relatively small sample (*n* = 90), it should be interpreted with caution. Nonetheless, it opens an intriguing avenue for future research to investigate potential biological mechanisms linking physical activity and smartphone addiction, ideally through replication and mechanistic validation in larger, diverse samples.

### 4.1 Limitations

This revised review shares several limitations with the original. Most studies relied on self-reported data, which is susceptible to recall bias and estimation errors. None could confirm whether smartphone addiction represents a clinical dysfunction through standardized clinical evaluation. Although the Diagnostic and Statistical Manual of Mental Disorders, Fifth Edition (DSM-5; American Psychiatric Association, 2013) criteria for behavioral addictions inform many tools used (e.g., Smartphone Addiction Scale-Short Version), they remain self-report measures and do not provide clinical diagnoses. This restricts the ability to differentiate between at-risk use and clinically significant impairment. Future research should work with clinical professionals to establish behavioral thresholds and support the development of formal diagnostic criteria as the field progresses.

Using volunteer samples introduces self-selection bias, and only 9 out of 16 studies clearly stated their hypotheses. Another key limitation is that 14 out of the 16 included studies employed cross-sectional designs, which restricts the ability to draw causal inferences. While these studies offer important correlational insights, they cannot determine the directionality or confirm the efficacy of physical activity as an intervention for smartphone addiction. Only one randomized controlled trial and one longitudinal study were available in this update, which limited the review's capacity to compare study types. Future systematic reviews should aim to include a greater number of experimental and longitudinal studies (if available) to enable sensitivity analyses and enhance causal interpretation.

All 16 included studies were conducted in Asia (China, Turkey, Pakistan, and Thailand), with no representation from Western or other cultural contexts. This limits the external validity of the findings, as cultural, educational, and socioeconomic factors may influence both smartphone use and physical activity patterns. Consequently, the conclusions drawn in this review primarily apply to university students in Asian contexts. Future studies from diverse regions are necessary to confirm whether these patterns hold across different cultural settings. Methodological issues persist, including the failure to distinguish between hedonic and utilitarian smartphone use and the tendency to equate excessive use solely with screen time. Future research should prioritize objective measurements, qualitative validation, longitudinal designs, and the exploration of biological markers to deepen our understanding of smartphone addiction. Where statistical reporting is sufficiently detailed and designs are comparable, future meta-analyses could provide stronger quantitative insights into the strength and variability of the relationship between physical activity and smartphone addiction. While the available effect sizes were converted to Cohen's d for interpretative clarity, variability in reporting standards and methodological heterogeneity limited formal statistical synthesis. Inconsistent use of smartphone addiction and physical activity measurement tools and heterogeneity in effect size reporting reduced comparability. Future research should adopt standardized, validated instruments and consider objective tools, such as screen-time or fitness trackers, to mitigate bias and enhance the potential for synthesis.

## 5 Conclusions

This updated systematic literature review supports a relatively consistent inverse relationship between physical activity and smartphone addiction among university students, extending on our previous findings. Emerging studies have identified key mediators (self-control, self-esteem, psychological resilience) and moderators (exercise intensity, type of smartphone use). Novel biological insights reveal a connection to gut microbiota, introducing an interdisciplinary aspect. The findings suggest that physical activity may serve as a protective factor through psychological and physiological pathways, highlighting smartphone addiction's multifactorial nature. Future research should employ longitudinal or experimental designs, use more objective measurements, and integrate qualitative or biological data into their protocol to gain a deeper understanding of how physical activity helps mitigate the risks associated with smartphone addiction. Finally, more Western studies are needed because smartphone addiction is a general worldwide concern.

## Declaration of generative AI in scientific writing

While preparing this work, the author(s) used AI to check for grammar and spelling mistakes. After using this tool/service, the authors reviewed and edited the content as needed and take full responsibility for the content of the published article.

## CRediT authorship contribution statement

**Neha Pirwani:** Writing – original draft, Methodology, Investigation, Conceptualization. **Attila Szabo:** Writing – review & editing, Supervision, Methodology, Investigation, Conceptualization.

## Funding

The authors have not received any funding for writing this review.

## Declaration of competing interest

The authors declare that they have no known competing financial interests or personal relationships that could have appeared to influence the work reported in this paper.

## Data Availability

No data was used for the research described in the article.

## Appendix A Supplementary data

Supplementary material Image 1
