# Supplementary material for: One-year update on physical activity and smartphone addiction in university students: A systematic review of novel research
Source: Prev Med Rep. 2025 Jul 20;57:103178. doi: 10.1016/j.pmedr.2025.103178 (PMC12304691; doi:10.1016/j.pmedr.2025.103178)
Supplement: Supplementary file 1 — Supplementary material [file mmc1.docx]

**Supplementary Table 4**

*Reference List of Studies Included in the Systematic Review on Smartphone Addiction and Physical Activity in University Students (Published 2024–2025)*

Aligül, B., & Tolukan, E. (2024). The relationship of smartphone addiction with motivation to participate in physical activity: A study in sports sciences faculty students. *Akdeniz Spor Bilimleri Dergisi, 7*(2), 320–329. https://doi.org/10.38021/asbid.1476185

Anwar, R., Ali, A., & Ahmed, F. (2024). Varsity sports to combat smartphone addiction among university students. *Journal of Development and Social Sciences, 5*(1), 293–301. https://doi.org/10.47205/jdss.2024(5-i)27

Ke, Y., Liu, X., Xu, X., He, B., Wang, J., Zuo, L., Wang, H., & Yang, G. (2024). Self-esteem mediates the relationship between physical activity and smartphone addiction of Chinese college students: A cross-sectional study. *Frontiers in Psychology, 14*. https://doi.org/10.3389/fpsyg.2023.1256743

Kumban, W., Cetthakrikul, S., & Santiworakul, A. (2025). Smartphone addiction, screen time, and physical activity of different academic majors and study levels in university students. International Journal of Environmental *Research and Public Health, 22*(2), 237. https://doi.org/10.3390/ijerph22020237

Lai, C., Cai, P., Liao, J., Li, X., Wang, Y., Wang, M., Ye, P., Chen, X., Hambly, B. D., Yu, X., Bao, S., & Zhang, H. (2025). Exploring the relationship between physical activity and smartphone addiction among college students in Western China. *Frontiers in Public Health, 13*. https://doi.org/10.3389/fpubh.2025.1530947

Liang, Y., & Tang, Y. (2025). The biomechanical influence of physical exercise on mobile phone addiction in college students: Mediating and moderating roles. *Molecular & Cellular Biomechanics, 22*(1), 787–787. https://doi.org/10.62617/mcb787

Liu, Y., Tan, D., Wang, P., Xiao, T., Wang, X., & Zhang, T. (2024). Physical activity moderated the mediating effect of self-control between bullying victimization and mobile phone addiction among college students. *Scientific Reports, 14*(1). https://doi.org/10.1038/s41598-024-71797-2

Meng, S., Qi, K., Shen, P., Zhang, M., Zhang, Y., Onyebuchi, N., Zhan, G., Wei, F., Tong, W., Han, Y., & Ge, X. (2025). The effects of mobile phone addiction on learning engagement of Chinese college students – the mediating role of physical activity and academic self-efficacy. *BMC Public Health, 25*(1). https://doi.org/10.1186/s12889-024-21250-w

Su, Y., Li, H., Jiang, S., Li, Y., Li, Y., & Zhang, G. (2024). The relationship between nighttime exercise and problematic smartphone use before sleep and associated health issues: A cross-sectional study*. BMC Public Health, 24*(1). https://doi.org/10.1186/s12889-024-18100-0

Wang, F. (2025). Effects of physical activity on mobile phone addiction among university students: The mediating roles of self-control and resilience*. Frontiers in Psychology, 16*. https://doi.org/10.3389/fpsyg.2025.1503607

Wang, J., Liu, X., Xu, X., Wang, H., & Yang, G. (2024a). The effect of physical activity on sleep quality among Chinese college students: The chain mediating role of stress and smartphone addiction during the COVID-19 pandemic. *Psychology Research and Behavior Management, 17*, 2135–2147. https://doi.org/10.2147/PRBM.S462794

Wang, Q., Chen, Y., & Li, L. (2024b). Effects of physical activity and self-control on mobile phone addiction in college students: A cross-lagged study in China. *Frontiers in Psychology, 15*. https://doi.org/10.3389/fpsyg.2024.1417379

Yin, Z., Yang, C., Liu, T., Yu, J., Yu, X., Huang, S., & Zhang, Y. (2024). The relationship between physical activity and sleep quality among college students: The chain-mediating effects of self-control and mobile phone addiction. *PLOS ONE, 19*(12), e0315930. https://doi.org/10.1371/journal.pone.0315930

Zeren, M., Eroğlu, R., Şahin, N. Y., Özdemir, K., Dönmez, B., & Karakurum, E. (2021). Does smartphone addiction impair maximal exercise capacity in young adults? *Journal of Basic and Clinical Health Sciences, 8*(1), 46–54. https://doi.org/10.30621/jbachs.1248796

Zhang, K., Guo, H., Zhang, X., Yang, H., Yuan, G., Zhu, Z., Lu, X., Zhang, J., Du, J., Shi, H., Jin, G., Ren, J., Hao, J., Sun, Y., Su, P., & Zhang, Z. (2024). Effects of aerobic exercise or Tai Chi Chuan interventions on problematic mobile phone use and the potential role of intestinal flora: A multi-arm randomized controlled trial. *Journal of Psychiatric Research, 170*, 394–407. https://doi.org/10.1016/j.jpsychires.2024.01.012

Zhu, W., Liu, J., Lou, H., Mu, F., & Li, B. (2024). Influence of smartphone addiction on sleep quality of college students: The regulatory effect of physical exercise behavior. *PLOS ONE, 19*(7), e0307162. https://doi.org/10.1371/journal.pone.0307162
